# Clinical application for the preservation of phospho-proteins through in-situ tissue stabilization

**DOI:** 10.1186/1477-5956-8-61

**Published:** 2010-11-22

**Authors:** C Bart Rountree, Colleen A Van Kirk, Hanning You, Wei Ding, Hien Dang, Heather D VanGuilder, Willard M Freeman

**Affiliations:** 1 Department of Pediatrics, Penn State Hershey Medical Center, 500 University Drive, Hershey, PA 17033; 2 Department of Pharmacology, Penn State College of Medicine, 500 University Drive, Hershey, PA 17033

## Abstract

**Background:**

Protein biomarkers will play a pivotal role in the future of personalized medicine for both diagnosis and treatment decision-making. While the results of several pre-clinical and small-scale clinical studies have demonstrated the value of protein biomarkers, there have been significant challenges to translating these findings into routine clinical care. Challenges to the use of protein biomarkers include inter-sample variability introduced by differences in post-collection handling and *ex vivo *degradation of proteins and protein modifications.

**Results:**

In this report, we re-create laboratory and clinical scenarios for sample collection and test the utility of a new tissue stabilization technique in preserving proteins and protein modifications. In the laboratory setting, tissue stabilization with the Denator Stabilizor T1 resulted in a significantly higher yield of phospho-protein when compared to standard snap freeze preservation. Furthermore, in a clinical scenario, tissue stabilization at collection resulted in a higher yield of total phospho-protein, total phospho-tyrosine, pErkT202/Y204 and pAktS473 when compared to standard methods. Tissue stabilization did not have a significant effect on other post-translational modifications such as acetylation and glycosylation, which are more stable *ex-vivo*. Tissue stabilization did decrease total RNA quantity and quality.

**Conclusion:**

Stabilization at the time of collection offers the potential to better preserve tissue protein and protein modification levels, as well as reduce the variability related to tissue processing delays that are often associated with clinical samples.

## Background

Recent advances in proteomic technologies have spurred a number of reports examining distinct alterations in protein expression [1,2] or modification [3-6] that are associated with, or can classify, disease states in human patients. Although these biomarker studies provide important analytical and diagnostic tools, a challenge for translational research is the transition of findings from the controlled laboratory environment to the clinical setting, where variation in tissue acquisition and handling practices can introduce significant data variability. This variation can confound data analysis and interpretation, and in turn, impact patient diagnosis and prognosis [7]. Combined with clinical heterogeneity resulting from genetic, physiological, and environmental factors, which are typically controlled for in animal models implemented in the laboratory setting, technical variance introduced during tissue collection in the clinical research setting reduces the statistical and classification power of translational studies. This is especially true regarding measurements of protein abundance and modification (e.g. phosphorylation). Standardization procedures have been proposed for plasma and serum collection in biomarker studies [8] and technologies for sample preservation of plasma and serum have been developed [9]. While no standards currently exist for tissue collection, technical approaches to preserve proteins and reduce technical variance have been proposed [10].

Whether in the laboratory or clinical setting, variations in tissue retrieval and processing, and any delay in sample stabilization (e.g., cryopreservation, fixation) can dramatically alter the quantitative characteristics of the tissue proteome. As tissue protein biomarkers seek to make the transition from the laboratory to the clinic, a real obstacle is standardizing tissue sample collection and processing in and around the operating suite, where most clinical samples are obtained. Total protein amounts and post-translational modifications are rapidly impacted by post-collection enzymatic activity. For example, *ex vivo *protease and phosphatase activity is retained but does not reflect true physiological conditions. Artifacts resulting from this residual activity not only increase inter-sample variability but also contribute to quantitation inaccuracies, particularly in measures of dynamic modification states of a given protein (e.g., phosphorylation) [11-13]. Traditional approaches to preserving tissues, including freezing and chemical fixation, require the availability of dry ice and chemicals in the operating suite. In the clinical environment, the primary focus of the surgical team is on the patient. In this setting, several hours may elapse from the time of tissue collection to preservation, depending on the time of collection and the availability of personnel [7].

A recent report by Svensson et al. demonstrates the success of rapid tissue stabilization in improving proteomic analyses. Using an approach combining heat and pressure inactivation of enzymes *ex vivo*, samples can be rapidly stabilized (< 1 minute) to prevent protein degradation and loss of post-translational modifications in tissue samples [10]. This technique does not utilize dry ice or chemicals and reduces sample complexity by preventing the formation of abundant protein degradation fragments and maintains modified species for up to two hours at room temperature. More recently, several papers have highlighted this technique for proteomic and peptidomic stabilization [14-17]. In this report, we seek to address unanswered questions related to this technique such as: 1) the stabilization of nucleic acid in combination with the tissue proteome, 2) an analysis of the entire phospho-proteome in addition to specific phospho-proteins such as Erk and Akt, 3) a detailed analysis of multiple clinical scenarios including a two hour room-temperature and two hour 4°C incubation, and 4) an analysis of non-phosphorylation protein modification such as glycosylation. The adoption of this preservation technique in clinical tissue sample collection has the potential to mitigate the deleterious effects of unavoidable delays in sample preservation, which would allow for improved biomarker screening. The purpose of the present study was to determine whether this commercially available method of rapid tissue stabilization increases phospho-protein stability and improves quantitation in tissue samples reflecting laboratory and clinically-relevant scenarios of sample collection.

## Results

**Figure 1:**
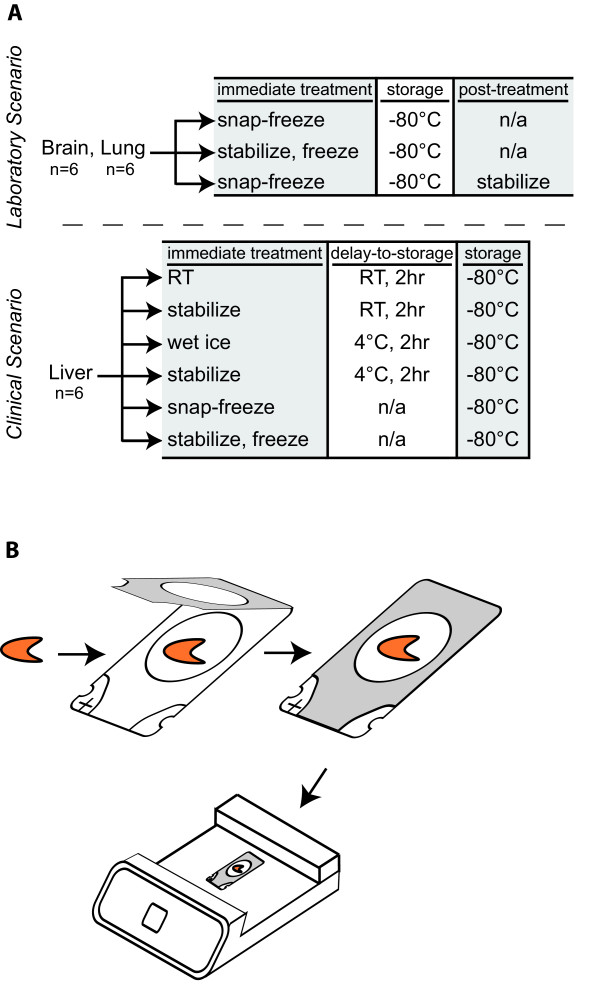
**Tissue treatment groups and stabilization procedure**. (A) Brain and lung samples were prepared according to three scenarios typical of the laboratory setting (n = 6/scenario). For liver samples the preparations were expanded to six different conditions potentially encountered in the clinical setting/operating room (n = 6/scenario). (B) Dissected tissue is placed in a hinged Maintainor cassette. The tissue is placed on the flexible Teflon window in the center of the cassette and the cassette is closed. Once placed in the Stabilizer T1 instrument, the air is removed from the cassette by vacuum and subjected to uniform heat and pressure, as described in the Methods.

In order to capture the different potential laboratory and clinical scenarios, we organized animals into groups as depicted in Figure 1A. For the laboratory setting, following tissue dissection, samples were: 1) snap frozen immediately for storage at -80°C, 2) stabilized immediately prior to storage at -80°C, or 3) snap frozen immediately prior to storage at -80°C and stabilized after removal from storage (Figure 1A). To reflect the broader array of clinically-relevant tissue handling scenarios, liver samples were dissected into six pieces as follows: 1) maintained at room temperature for two hours, and stored at -80°C; 2) stabilized immediately, maintained at room temperature for two hours, and stored at -80°C; 3) maintained at 4°C for two hours, and stored at -80°C; 4) stabilized immediately, maintained at 4°C for two hours, and stored at -80°C; 5) snap frozen immediately ("best-case scenario" control) and stored at -80°C; or 6) stabilized immediately, snap frozen, and stored at -80°C (Figure 1A).

**Figure 2:**
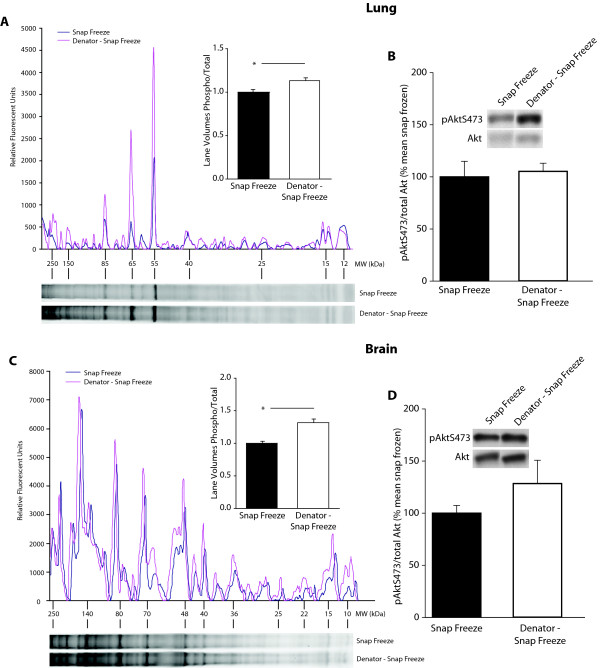
**Preservation of phospho-protein levels in lung and brain samples in laboratory scenarios**. SDS-PAGE separated lung (A) and brain (C) total and phospho-protein levels were determined by SyproRuby (total) and ProQ Diamond (phospho) staining, respectively. In both tissues stabilization resulted in increased phospho- to total protein levels as compared to snap frozen control samples. Targeted phosphorylation was examined by immunoblotting using pAktS473 as an example protein. In both the lung (B) and brain (D), pAktS473 to total Akt ratios were higher in the stabilized tissue samples, but did not reach statistical significance. Data are presented as mean ± S.E.M., n = 6/group (* p < 0.05, one way ANOVA with SNK).

In the "laboratory setting" model, immediate stabilization of brain and lung tissue following dissection resulted in higher phospho-protein levels compared to snap frozen controls. Using automated densitometry methods, total lane volumes were determined for both the phospho-protein and total protein stains, and ratios of phospho- to total protein were calculated. In lung tissue, phospho-protein to total protein ratios were significantly greater in stabilized samples compared to snap frozen controls and snap frozen then stabilized samples (1.13 ± 0.03 vs. 1.0 ± 0.03 and 0.98 ± 0.03 respectively, p = 0.011, n = 6/group) (Figure 2A). No statistically significant difference was observed between snap frozen controls and snap frozen then stabilized samples (data not shown). In brain, the average ratio of phospho-protein to total protein was significantly greater in stabilized samples compared to snap frozen controls and snap frozen then stabilized samples (1.31 ± 0.05 vs. 1.0 ± 0.03 and 1.0 ± 0.04, respectively; p < 0.001, n = 6/group) (Figure 2C). Once again, no statistically significant difference was observed between snap frozen controls and snap frozen then stabilized samples (data not shown). These data demonstrate that significantly higher levels of total phosphorylated proteins are detected in stabilized samples isolated from both brain (Figure 2C) and lung (Figure 2A) than in snap frozen tissue samples. This is true for both small and large molecular weight phospho-proteins. Graph insets illustrate the average phospho-protein to total protein ratios scaled to the respective control means. To further analyze the phospho-protein levels, pAktS473 was examined by immunoblotting in both the lung and brain. While higher pAktS473/total Akt ratios were observed in stabilized tissue versus snap frozen controls, this difference did not reach statistical significance (Figures 2B and 2D respectively).

**Figure 3:**
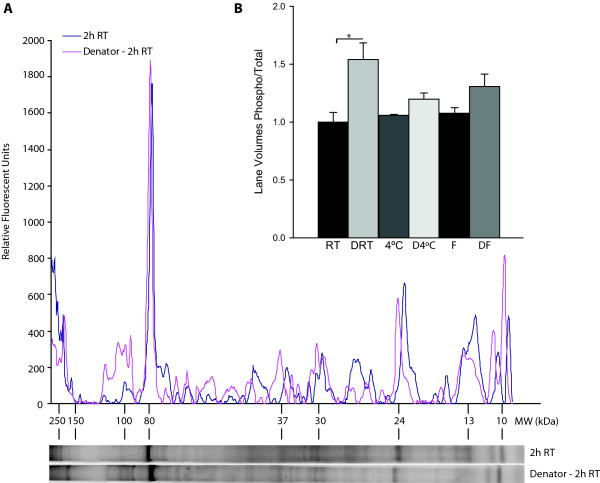
**Preservation of phospho-protein levels in liver samples in clinical scenarios**. (A) SDS-PAGE separated liver total and phospho-protein levels were determined by SyproRuby (total) and ProQ Diamond (phospho) staining, respectively. (B) Stabilization, followed by 2 hours at room temperature prior to cryostorage, resulted in increased phospho- to total protein levels as compared to non-stabilized control samples and samples stored at 4°C for 2 hours. Data are presented as mean ± S.E.M., n = 3/group (* p < 0.05, un-paired t-test). F - snap frozen; DF - Denator, snap freeze; FD - snap freeze, Denator; RT - room temperature; DRT - Denator, room temperature 2 hrs; 4°C - wet ice 2 hrs; D4°C - Denator, wet ice 2 hrs.

Although the laboratory setting provides a well controlled environment for tissue collection, the clinical setting is a more challenging venue. To create an accurate recapitulation of the clinical setting, we first determined typical tissue collection times in the clinic. In our research hospital setting, laboratory staff (CBR) acted to transport liver tissue resected from patients directly from the OR to pathology for initial processing, and then to the laboratory for cryopreservation as part of an IRB approved protocol. The average time from tissue collection to cryopreservation, with staff immediately available for tissue transport in the OR at the time of initial retrieval, was 66 ± 14 minutes (n = 4). Even in this ideal setting, there is an increased probability that specific disease biomarkers, especially phospho-proteins, may be significantly altered, thereby confounding the analysis and interpretation of any biochemical experiments. To model the clinical environment, "clinical control" liver samples were left at room temperature or on wet ice (4°C) for two hours prior to cryopreservation. These samples were compared to tissue stabilized immediately and left at room temperature for two hours. Total phospho-protein levels in stabilized samples were approximately 50% higher than in samples incubated at room temperature or 4°C (1.5 ± 0.14, Denator, 2 hrs RT; 1.0 ± 0.08, RT 2 hrs, p < 0.02, n = 3/group) (Figure 3A and 3B).

**Figure 4:**
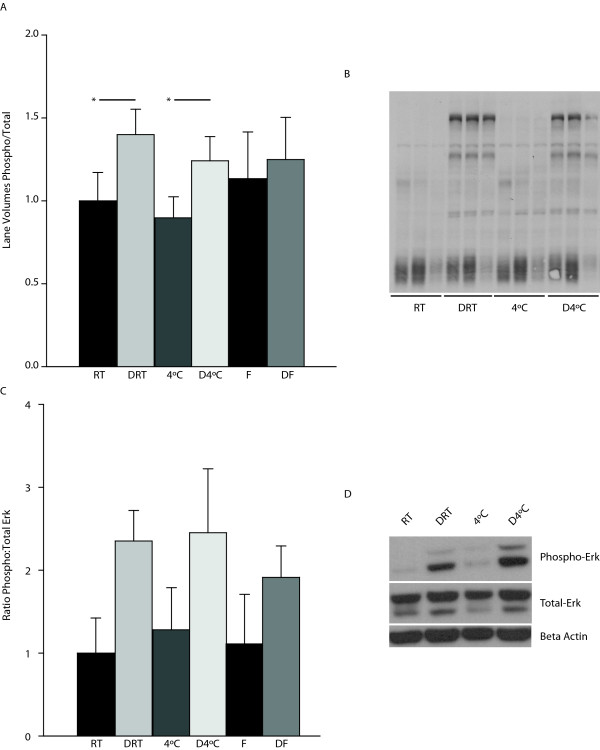
**Preservation of total phosphorylated tyrosine and phospho-Erk**. (A) Stabilization, followed by 2 hours at room temperature or 4°C, resulted in increased phospho- to total protein levels as compared to non-stabilized samples. Data are presented as mean ± S.E.M., n = 6/group (*p < 0.05, paired t-test). (B) Representative blot of phosphorylated tyrosine in immediately stabilized samples followed by 2 hours at room temperature or 4°C compared to control non-stabilized samples. (C) Rapid stabilization increased the amount of phosphorylated Erk detected. Data are presented as means ± S.E.M., n = 5/group. (D) Representative blots of phosphorylated Erk and total Erk in samples stabilized followed by incubation for 2 hours at room temperature or 4°C and control samples. F - snap frozen; DF - Denator, snap freeze; FD - snap freeze, Denator; RT - room temperature; DRT - Denator, room temperature 2 hrs; 4°C - wet ice 2 hrs; D4°C - Denator, wet ice 2 hrs.

To more specifically examine preservation of phosphorylation, pan phospho-tyrosine levels and specific phosphorylated proteins were examined. Total phospho-tyrosine levels were found to be significantly higher in tissue that was immediately stabilized when compared to tissue incubated at room temperature (1.4 ± 0.15, Denator, 2 hrs RT vs. 1.0 ± 0.17, RT 2 hrs; p < 0.05; n = 6/group) (Figure 4A and 4B). Similarly, immediate stabilization followed by two hours at 4°C preserved a significantly higher amount of phospho-tyrosine in comparison to samples incubated at 4°C (1.2 ± 0.15, Denator, 2 hrs 4°C vs. 0.9 ± 0.13, 4°C 2 hrs; p < 0.05, n = 6/group). Erk is a major cell signaling protein in the MAPKinase pathway that is activated by phosphorylation on threonine 202 and tyrosine 204. Although there was a marked increase in total phospho-Erk following stabilization in both room temperature and 4°C paired groups, the overall effect was not statistically significant (Figure 4C and 4D).

**Figure 5:**
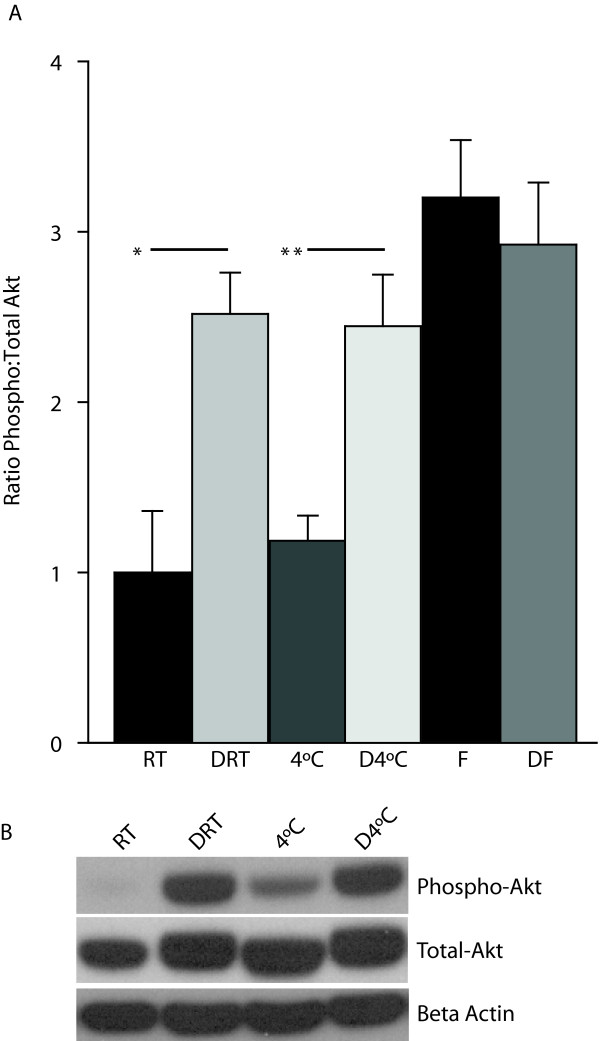
**Phopsho-Akt levels are significantly higher in stabilized samples**. (A) Stabilization of tissue samples followed by incubation at either room temperature or at 4°C yielded greater pAktS473 levels compared to non-stabilized samples. Stabilization resulted in pAktS473 yields comparable to immediate cryopreservation. Data are presented as mean ± S.E.M., n = 5/group (*p < 0.05, **p < 0.005, paired t-test). (B) Representative blots of pAktS473, total Akt, and beta-Actin in samples rapidly stabilized followed by incubation for 2 hours at room temperature or 4°C and control samples stored at room temperature or 4°C for two hours without stabilization. F - snap frozen; DF - Denator, snap freeze; FD - snap freeze, Denator; RT - room temperature; DRT - Denator, room temperature 2 hrs; 4°C - wet ice 2 hrs; D4°C - Denator, wet ice 2 hrs.

Akt is a second intracellular signal mediator activated by the PI3Kinase pathway and is phosphorylated on serine (S473) residues rather than on tyrosine. To examine the effects of immediate stabilization on the preservation of serine/threonine phosphorylation, relative amounts of phospho-Akt were determined. Immediate stabilization followed by two hours at room temperature preserved significantly higher levels of pAktS473 as compared to non-stabilized samples (2.5 ± 0.24, Denator, 2 hrs RT; 1.0 ± 0.36, RT 2 hrs, p < 0.05, n = 5/group) (Figure 5A and 5B). Similarly, immediate stabilization followed by two hours at 4°C yielded higher levels of pAktS473 as compared to non-stabilized samples incubated at 4°C for two hours alone (2.45 ± 0.30, Denator, 2 hrs 4°C; 1.19 ± 0.15, 4°C 2 hrs, p < 0.005). This indicates that immediate stabilization preserves phospho-Akt comparable to immediate cryopreservation regardless of delays of 2 hrs at room temperature and 4°C (Figure 5). These results further demonstrate that integration of rapid tissue stabilization in the operating suite can preserve phospho-protein integrity.

**Figure 6:**
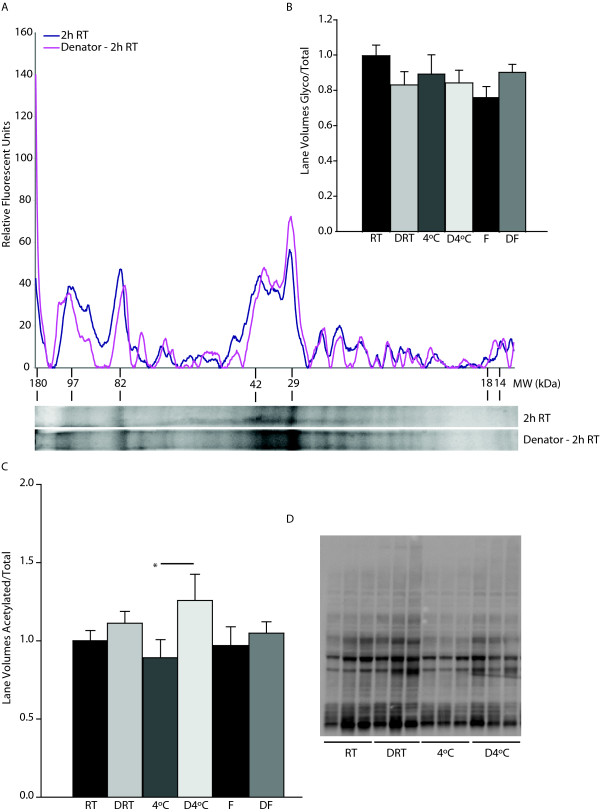
**Effects of rapid stabilization on protein glycosylation and acetylation**. (A) SDS-PAGE separated liver total protein and glyco-protein levels were determined by SyproRuby (total) and ProQ Emerald (glyco) staining, respectively. (B) Stabilization had no effect, either positive or negative, on the ratio of glyco:total protein. Data are presented as mean ± S.E.M., n = 3/group. (C) Total acetylated lysine was assessed, and stabilization followed by 2 hours at 4°C resulted in increased acetylated protein to total protein levels as compared to non-stabilized samples stored at 4°C for 2 hours. Data are presented as mean ± S.E.M., n = 6/group (*p < 0.05, paired t-test). (D) Representative blot of total acetylated lysine in immediately stabilized samples followed by 2 hours at room temperature or 4°C prior to cryostorage and control samples stored at room temperature or 4°C for 2 hours. F - snap frozen; DF - Denator, snap freeze; FD - snap freeze, Denator; RT - room temperature; DRT - Denator, room temperature 2 hrs; 4°C - wet ice 2 hrs; D4°C - Denator, wet ice 2 hrs.

Although immediate stabilization has been shown to be useful in the preservation of phospho-proteins, little is known concerning its effects on the preservation of other protein modifications, such as glycosylation and acetylation. Interestingly, there was no statistically significant difference between the amounts of total glycosylated-protein levels in stabilized vs. non-stabilized tissues (Figure 6A and 6B) (line graph for other four conditions not shown). No significant differences were observed in acetylated protein when comparing non-stabilized and stabilized tissues, with the exception of tissue that was immediately stabilized then incubated at 4°C when compared to tissue incubated at 4°C only (1.3 ± 0.17, Denator, 2 hrs 4°C; 0.89 ± 0.12, 4°C 2 hrs; p < 0.05; n = 6/group) (Figure 6C and 6D).

**Figure 7:**
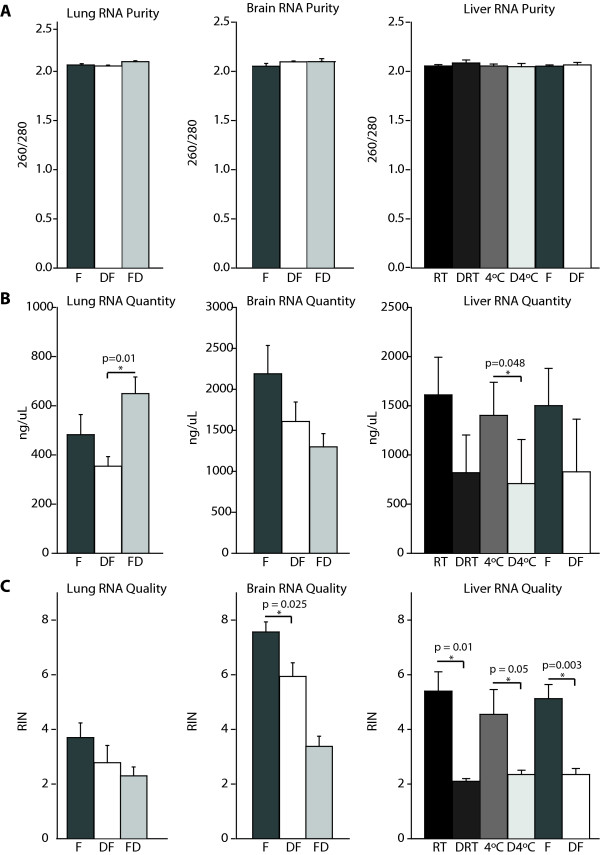
**RNA quantity and integrity following immediate stabilization**. (A) Relative RNA purity, defined as the ratio of absorbance at 260 and 280 nm, was determined by NanoDrop. A ratio of 2.0 is indicative of pure RNA. No significant differences were apparent across the treatment groups in any tissue. (B) RNA quantity was determined by NanoDrop. No significant differences were seen in relative RNA quantity in the brain regardless of treatment. In lung, when stabilization followed cryopreservation, RNA quantity increased when compared to samples immediately stabilized followed by cryopreservation (*p < 0.05, Kruskal-Wallis one way ANOVA on Ranks). In liver tissue, stabilization decreased total RNA quantity when compared to similar samples incubated at room temperature, 4°C, and immediately cryopreserved samples. (C) RNA quality, determined by relative amounts of degraded vs. intact RNA, was measured by Bioanalyzer and assigned an RNA integrity number (RIN). RINs range from 10 (intact) to 2 (degraded) with an average RIN being 7. While there were no statistically significant differences seen in lung tissue, in brain, RNA quality significantly decreased with immediate stabilization prior to cryopreservation compared to immediately cryopreserved samples. In liver, immediate stabilization significantly decreased RNA quality when compared to similar samples incubated at room temperature, 4°C, and immediately cryopreserved samples. Data are presented as mean ± S.E.M., n = 5-6/group brain and lung, n = 4/group liver (* p < 0.05, unpaired t-test). F - snap frozen; DF - Denator, snap freeze; FD - snap freeze, Denator; RT - room temperature; DRT - Denator, room temperature 2 hrs; 4°C - wet ice 2 hrs; D4°C - Denator, wet ice 2 hrs.

When examining the effects of immediate stabilization on RNA, overall RNA purity was found to be comparable across all treatment groups within each tissue type regardless of Denator use (Figure 7A). No significant difference in total brain RNA quantity was observed if stabilization preceded immediate cryopreservation. In lung, immediate cryopreservation prior to stabilization significantly increased RNA quantity when compared to samples immediately stabilized followed by cryopreservation (p < 0.05). In liver tissue, stabilization decreased total RNA quantity when compared to similar samples incubated at room temperature, 4°C (1401.9 ± 168.13, 2 hrs 4°C vs. 708.2 ± 224.37, Denator, 2 hrs 4°C, p < 0.05, n = 4/group) and samples immediately snap frozen without a stabilization step (Figure 7B). Although there was no significant difference between treatment groups in terms of RNA quality in lung tissue, brain RNA quality was significantly higher in the immediate snap freeze control samples without stabilization compared to the immediately stabilized samples (7.6 ± 0.37 snap freeze vs. 5.9 ± 0.50 Denator, freeze, p < 0.05, n = 5-6/group) (Figure 7C). In liver tissue, immediate stabilization decreased total RNA quality compared to samples without stabilization treatment (5.4 ± 0.71, 2 hrs RT vs. 2.1 ± 0.10, Denator, 2 hrs RT, p < 0,05, n = 4/group), 4°C (4.6 ± 0.91, 2 hrs 4°C vs. 2.4 ± 0.16, Denator, 2 hrs 4°C, p < 0.05, n = 4/group), and to samples immediately cryopreserved without the stabilization step (5.1 ± 0.52 snap freeze vs. 2.4 ± 0.2 Denator, freeze, p < 0.005, n = 4/group) (Figure 7C), demonstrating significant degradation of RNA in all stabilization groups compared to their paired, non-stabilized controls.

## Discussion

Biospecimens have been utilized in pathology research for more than a century, and currently, many institutional pathology departments comprise the largest sample repositories [18]. The quality and utility of these biospecimen collections is often questionable, however, due to the variations in tissue handling processes, which may be subject to the same delays as cryopreservation. Although routine formalin fixation and paraffin embedding procedures preserve the structure and architecture of specimens, they do not guarantee the preservation of molecular integrity, which is critical to downstream analyses.

Because clinical activities necessarily focus on the patient, samples are often left to be affected by factors including poor tissue handling, warm ischemia time, fixation, and storage conditions [7]. It is important to improve standard protocols for tissue collection. This includes adequate stabilization that preserves the molecular integrity of the sample, appropriate storage conditions to optimize biospecimen stability, biomolecule extraction techniques to improve sample quality, and the active involvement of pathologists in the quality control of biospecimen collection [19]. In general, stabilization techniques, which do not use chemicals that may interfere with downstream analyses and which are simple and quick enough to be routinely used, are preferred.

Delays in cryopreservation in translational research include operating suite processing, transport to surgical pathology, and transport of tissue to the laboratory for processing. In the ideal setting, where research staff (CBR) waited in the operating suite to retrieve the sample directly from the surgical team, personally transported tissue from the operating suite to pathology, and then transported the research tissue directly to the laboratory for cryopreservation, it took more than an hour. Loss of quality can occur up front, as biospecimens may sit unprocessed for many hours before being fixed, and more often than not, there is no standard protocol or requirement to document how the tissue is handled. Without this knowledge, a researcher has no way of determining whether the results of downstream molecular experimentation reflect the effects of tissue handling or the *in vivo *state. The tissue stabilization process presented here takes less than 30 seconds and provides a simple and effective mechanism for rapid stabilization of clinical tissues that more easily preserves protein biomarkers over alternative preservation methods such as snap freezing. Additionally, using this method, stabilized samples can be kept at room temperature for several hours prior to cryopreservation or tissue analysis without decreases in phospho-protein content. This provides investigators with a tool that increases consistency of samples collected in the operating suite for proteomic analysis. Immediate stabilization also did not alter the levels of protein modifications such as glycosylation and acetylation, which are more resistant to *ex-vivo *degradation [20,21]. This finding serves as a negative control, suggesting that stabilization does not introduce any variability or alter such modifications, making examination of a wide variety of protein modifications possible. However, RNA quantity and quality is significantly reduced as a result of this stabilization technique, indicating a potential limitation for use when examining the tissue proteome only. By using the Stabilizor T1 in the operating room or pathology suite, the clinical team may continue to focus on patient care and rapidly stabilize a part of biopsy samples to significantly decrease phospho-protein degradation. A number of diagnostic biomarker development efforts have focused on protein expression [1,2]. Recent studies have demonstrated that protein modification levels can be used for diagnostic and prognostic purposes [3,5,22,6]. Usage of the Stabilizor T1 would potentially allow for more accurate biochemical diagnostic tests, especially those designed to monitor phospho-protein levels. This process requires one piece of instrumentation, is rapid (< 1 min), and prevents decreases in phospho-protein content with short-term storage at room temperature. Future studies will test implementation of this stabilization process in the operating suite to improve the quality of tissue specimen collection.

## Conclusions

Tissue stabilization at collection offers the potential to more accurately preserve tissue protein and protein modification levels, such as phosphorylation, and reduce variability related to tissue processing delays.

## Materials and methods

### Animals

Three month old male C57BL6/J mice acquired from Jackson Laboratory (Bar Harbor, ME) were housed four per cage in solid-bottom cages in the Hershey Center for Applied Research animal facility, and maintained in a temperature-controlled environment on a 12/12 hour light/dark cycle with free access to water and food (Harlan Teklad irradiated mouse diet 7912, Madison, WI). All procedures were conducted in compliance with Penn State University guidelines for the use of laboratory animals and approved by the Institutional Animal Care and Use Committee.

### Tissue Processing

Immediately following sacrifice, brain, lung and liver tissue was rapidly dissected. Brain and lung tissue were treated in three ways to model the standard controlled laboratory research setting (n = 6/group): 1) snap frozen immediately on dry ice prior to storage at -80°C and processed for biochemical analyses after thawing normally, 2) stabilized immediately prior to storage at -80°C and processed after thawing, or 3) snap frozen immediately on dry ice prior to storage at -80°C and then stabilized after removal from storage and before biochemical analyses (Figure 1A). Liver samples (n = 6/group) were dissected into six pieces of roughly equal size that were treated as follows: 1) snap frozen immediately on dry ice and stored at -80°C; 2) stabilized immediately, frozen on dry ice, and stored at -80°C; 3) stabilized immediately, maintained on wet ice (4°C) for two hours, and stored at -80°C; 4) stabilized immediately, maintained at room temperature for two hours, and stored at -80°C; 5) maintained on wet ice (4°C) for two hours, and stored at -80°C; or 6) maintained at room temperature for two hours, and stored at -80°C (Figure 1A).

### Tissue Stabilization

Tissue stabilization was conducted using Stabilizor T1 instrumentation (Denator AB, Gothenburg, Sweden). Tissue samples were placed in inert polycarbonate/thermoplastic (Teflon-fluorinated ethylene propylene) Maintainor Cards (Denator AB), and air was removed by automated vacuum to minimize potential protein oxidation and maximize efficient heat transfer. Samples were then subjected to 5 mbar of pressure and heated to 95°C for 20 seconds to eliminate residual *ex vivo *biological activity (Figure 1B).

### Protein isolation and quantitation

Protein was extracted according to Denator AB-recommended procedures. Using an automated Retsch TissueLyser II bead mill (Qiagen, Inc., Germantown, MD) and stainless steel beads pre-chilled on dry ice, frozen tissue samples were homogenized at 15 Hz for one minute. A volume of 1% SDS, equal to 10 times the sample mass, was then added to each sample prior to tissue disruption at 15 Hz for one minute. The homogenization beads were then removed, and sample homogenates were incubated at 95°C for 10 minutes with shaking. During this incubation period, each sample was briefly sonicated (40 W, two seconds) at five minute intervals. Soluble protein was recovered by centrifuging tissue homogenates (10,000 × g, 4°C, 10 minutes) to pellet insoluble protein. Soluble protein concentrations were determined by BCA quantitation assay (Pierce, Rockford, IL).

### Total and phospho-protein analysis

Relative abundance of total and phospho-protein was determined by SDS-PAGE followed by SyproRuby (total) and ProQ Diamond (phospho) gel staining (Molecular Probes, Eugene, OR). For brain, lung, and liver samples, equal protein in equal volumes (30 μg in 12 μL) was separated by molecular weight using precast Criterion Tris-HCl 10.5%-14% acrylamide gradient gels (BioRad, Hercules, CA). Upon completion of electrophoresis, gels were fixed in 50% methanol/10% acetic acid and sequentially post-stained first with ProQ Diamond and SyproRuby according to manufacturer's instructions. Briefly, fixed gels were incubated with ProQ Diamond phospho-protein stain for 90 minutes at room temperature with gentle shaking. Following destaining with 20% acetonitrile/50 mM sodium acetate (pH 4.0), gels were imaged with a Typhoon 9410 fluorescent imager (GE Healthcare, Piscataway, NJ) with the following settings: green laser, 532 nm excitation, 555 nm (20 nm bandpass) emission. To image total protein abundance, gels were then co-stained with SyproRuby by overnight incubation at room temperature with gentle shaking. After destaining with 10% methanol/7% acetic acid, gels were imaged with the following settings: green laser, 532 nm excitation, 610 nm (30 nm bandpass) emission. Relative abundance of total protein and phospho-protein was quantitated by automated digital densitometry (1D gel analysis, ImageQuant TL software; Molecular Dynamics, Sunnyvale, CA).

### Immunoblot Analysis - Liver

To determine the relative abundance of phosphorylated tyrosine and acetylated lysine, 20 μg of soluble protein isolated from liver tissue was separated by molecular weight using precast Criterion Tris-HCl 10.5%-14% acrylamide gradient gels (BioRad, Hercules, CA) and transferred to polyvinylidene difluoride (PVDF) membranes (GE Healthcare). Following transfer, membranes were incubated in 0.1% w/v Ponseau S for five minutes, rinsed with ddH_2_O, and then imaged using a reflective scanner. Membranes were then blocked for one hour in 5% BSA in PBST (PBS and 0.1% Tween). Blots were incubated overnight at 4°C with primary antibodies (phospho-tyrosine and acetylated lysine mouse monoclonal antibodies, Cell Signaling, Danvers, MA) diluted in blocking solution. After washing with PBST, each blot was incubated with horseradish peroxidase (HRP)-conjugated mouse secondary antibody for two hours at room temperature. Signals were detected with ECL substrate (ThermoScientific, Rockford, IL), developed on film, and quantitated using automated digital densitometry (ImageQuant TL software, GE Healthcare).

To quantitate total and phosphorylated target proteins in the liver, 40 μg of soluble protein isolated from liver samples were separated by molecular weight using NuPage Bis-Tris 4-12% acrylamide precast gels (Invitrogen, Carlsbad, CA) and transferred to PVDF membranes (Invitrogen). After blocking with 5% nonfat milk in TBST buffer (20 mM Tris-HCl, pH 7.6, 136 mM NaCl, and 0.1% Tween-20) at room temperature (RT) for one hour, blots were incubated overnight at 4°C with primary antibodies (total Akt, phospho-Akt, total Erk, and phospho-Erk, all rabbit monoclonal antibodies, Cell Signaling, Danvers, MA; β-actin mouse monoclonal antibody, Sigma, St. Louis, MO) diluted in blocking solution. After washing with TBST, blots were incubated with horseradish peroxidase (HRP)-conjugated species appropriate secondary antibodies (Amersham Biosciences, Pittsburgh, PA) for one hour at room temperature. Signals were detected with ECL substrate (Amersham Pharmacia Biotech, Piscataway, NJ), developed on film and quantitated using automated digital densitometry.

### Immunoblot Analysis - Brain and Lung

To quantitate total and phosphorylated target proteins in the brain and lung, immunoblotting was performed as described above, using 30 μg of soluble lung protein and 15 μg of soluble brain protein.

### Total and glyco-protein analysis

Relative abundance of total and glyco-protein was determined by SDS-PAGE followed by SyproRuby (total) and ProQ Emerald (glyco) gel staining (Molecular Probes, Eugene, OR). Equal protein in equal volumes (30 μg in 12 μL) was separated by molecular weight using precast Criterion Tris-HCl 10.5%-14% acrylamide gradient gels. Subsequently, gels were fixed in 50% methanol and 5% acetic acid in ddH_2_O for 45 minutes two times with gentle agitation and then washed twice in 3% glacial acetic acid for 15 minutes. Following this, gels were incubated in an oxidizing solution (periodic acid dissolved in 3% acetic acid) for 30 minutes, and then washed three times in 3% glacial acetic acid. Gels were then placed into a light protected box and incubated with the ProQ Emerald staining solution for one hour with gentle agitation and imaged using a UV imager, EpiChemi Darkroom (UVP Bioimaging Systems, Upland, CA), at 302 nm. Following imaging, gels were rinsed with ddH_2_O and stained overnight with Sypro Ruby. Gels were then washed in 10% methanol and 7% acetic acid two times for 15 minutes, rinsed twice for five minutes in ddH_2_O, and scanned using a Typhoon 9410 fluorescent imager as described above.

### RNA isolation

Total RNA was isolated from each tissue using standard isolation methods[23-25]. Briefly, each tissue sample was homogenized in 500 μL cold Tri-Reagent (Sigma-Aldrich, St. Louis, MO) using an automated Retsch TissueLyser II bead mill (Qiagen, Inc., Germantown, MD) and stainless steel beads at 15 Hz for one minute. Sample volume was brought to 1 mL with additional Tri-Reagent and 0.1 mL BCP (Molecular Research Center, Inc., Cincinnati, OH) was added to separate phases. RNA was precipitated by adding 0.1 mL isopropanol to the isolated aqueous phase and incubating overnight. Following precipitation, RNA was purified using Qiagen RNeasy spin columns (Qiagen, Inc., Valencia, CA) and resuspended in RNase-free water.

### RNA quantitation and analysis

Both quality and quantity were evaluated using the RNA 6000 Nano LabChip with an Agilent 2100 Expert Bioanalyzer (Agilent, Palo Alto, CA) and NanoDrop ND100 (Nanodrop, Wilmington, DE) respectively. RNA integrity numbers (RINs), as measured by Bioanalyzer, are an accurate measure of RNA degradation with a range from 10 (intact) to 2 (degraded), and were therefore used to demonstrate RNA quality. Ratios of absorbance at 260 and 280 nm, as measured by Nanodrop, were used to measure RNA purity. Typically, a ratio of 2.0 is indicative of pure RNA.

### Statistical Analysis

All data was scaled to respective control means. Statistical analyses were performed using two-tailed t-tests, a one way ANOVA with a Student-Newman-Keuls (SNK) post hoc analysis, or a Kruskal-Wallis one way ANOVA for any data that failed normality testing, SigmaStat 3.5 (Systat Software, San Jose, CA).

## Competing interests

The authors declare that they have no competing interests.

## Authors' contributions

**CBR **performed initial conceptual design, designed all experiments, performed necroscopy for tissue dissection, performed data analysis, and wrote the manuscript. **CAVK **designed all experiments, executed experiments in brain and lung protein analysis and RNA analysis, preformed data analysis, and co-wrote the manuscript. **HY **executed experiments in liver sample preparation, protein analysis, and preformed densitrometry analysis. **WD **executed experiments in liver sample preparation and protein analysis, and preformed densitrometry and data analysis. **HD **executed experiments in liver protein analysis and RNA analysis and performed data analysis. **HDVG **designed experiments, executed experiments in brain and lung tissue processing and protein analysis, preformed data analysis, and assisted in initial manuscript draft. **WMF **Performed initial conceptual design, designed all experiments, assisted in necroscopy for tissue dissection, performed data analysis, and critical revisions of the manuscript. All authors have read and approved of the final manuscript.
